# *Streptococcus thermophilus* Attenuates Inflammation in Septic Mice Mediated by Gut Microbiota

**DOI:** 10.3389/fmicb.2020.598010

**Published:** 2020-12-15

**Authors:** Fu Han, Gaofeng Wu, Yijie Zhang, Haotian Zheng, Shichao Han, Xiaoqiang Li, Weixia Cai, Jiaqi Liu, Wanfu Zhang, Xiaowei Zhang, Dahai Hu

**Affiliations:** ^1^Department of Burns and Cutaneous Surgery, Xijing Hospital, Fourth Military Medical University, Xi’an, China; ^2^Key Laboratory of Tissue Repair and Regeneration of PLA and Beijing Key Research Laboratory of Skin Injury, Repair and Regeneration, First Hospital Affiliated to General Hospital of PLA, Beijing, China; ^3^BGI Education Center, University of Chinese Academy of Sciences, Shenzhen, China; ^4^Department of Obstetrics and Gynecology, Peking University Shenzhen Hospital, Shenzhen, China

**Keywords:** sepsis, inflammation, probiotics, Microbiome, *streptococcus thermophilus*

## Abstract

Sepsis is a life-threatening organ dysfunction condition caused by a dysregulated host response to infection and lack of effective treatment method. Supplementation of probiotics has emerged as a potential biotherapy for inflammatory diseases in recent years, but its role in protecting viscera against the damage caused by sepsis and the underlying mechanism is poorly understood. *Streptococcus thermophilus* 19 is one of the most well-studied probiotics, which is selected in this study among seven strains isolated from homemade yogurt due to its optimal ability of suppressing the inflammation response *in vitro*. It showed significant decrease in the expression of TNF-α, IL-1β, and IL-6 in the co-culture of *S. thermophilus* 19 and LPS-treated mouse macrophage. The effect of *S. thermophilus* 19 in mice and the response of mice gut microbiota were subsequently investigated. In LPS-induced septic mouse model, *S. thermophilus* 19 was highly resistant to LPS and exhibited significantly decreased expressions of inflammatory factors compared to LPS-treated mice. A MiSeq-based 16S rDNA sequence analysis revealed that the decrease of gut microbial diversity in mice intraperitoneally injected with 1 mg/ml LPS were mitigated by the administration of *S. thermophilus* 19. *Fusobacterium* significantly decreased during the development of sepsis and rose again after supplement strain 19, while *Flavonifractor* showed the opposite trend, which demonstrated these two genera were the key bacteria that may function in the mice gut microbiota for alleviation of LPS-induced inflammation reaction. To conclude, *S. thermophilus* 19 may be a potential candidate for novel biotherapeutic interventions against inflammation caused by sepsis.

## Introduction

Sepsis is a life-threatening organ dysfunction complication caused by severe host infection, which is responsible for up to 50% to 60% of burn injury deaths (Bai et al., 2018; Manning, 2018). Although our understanding of sepsis has increased substantially in recent years, effective treatment strategies have still not been developed. Even worse, the incidence of sepsis has increased annually. Therefore, new insights into the causes of sepsis are urgently needed.

The gut microbiota is a complex ecosystem consisting of trillions of bacteria that live in the digestive tracts of human beings and other animals (Glenwright et al., 2017). Growing evidence supports the key role of a healthy gut microbiota, which is established immediately after birth, in promoting and maintaining a balanced immune response (Maynard et al., 2012; Hand, 2016). Moreover, dysbiosis of the gut microbiota will lead to the dysregulation of various processes, which in turn develops as autoimmune conditions (Clemente et al., 2018). For instance, the presence or overabundance of *Bacteroides*, *Enterobacteriaceae*, and *Firmicutes* contribute to inflammatory disorders such as IBD. Additionally, metabolites from certain members of the gut flora may influence host signaling pathways, contributing to disorders such as colon cancer and obesity (Baumann-Dudenhoeffer et al., 2018). Sepsis is an extreme inflammation response throughout the entire body that releases the immune system chemicals into the bloodstream. For decades, the gut has been regarded as the motor of sepsis (Carrico et al., 1986), and it has recently been shown that a balanced gut microbiota has a protective role during systemic inflammation. Thus, we hypothesized that intestinal microbiota are also closely related to the cause and treatment of sepsis. Probiotics are live microbes that have beneficial effects on human and animal health when ingested in sufficient amounts (Isolauri et al., 2002). It has been reported that to the supplement of probiotics could treat or prevent a number of diseases by remediating gut microbial disorders, such as irritable bowel syndrome, hypercholesterolemia, gastritis, gut infection, parasitic infestation, hypersensitivity, and even cancers (Mombaerts et al., 1993; Doron and Snydman, 2015). The use of microbes as probiotics also hold potential for oral health in preventing and treating oral infections, dental plaque-related diseases, and periodontal diseases (Anusha et al., 2015; Lin et al., 2018). Furthermore, probiotics can alleviate inflammation associated with some human diseases by regulating the gut microbiota composition (Hwang et al., 2017; Wu et al., 2017). E. Wasilewska found that yogurt starter cultures of *Lactobacillus bulgaricus* 151 and *Streptococcus thermophilus* MK-10 had high anti-inflammatory potential in colitis.

In this study, we firstly used a co-culture system (bacterial strains and mouse macrophage cell RAW264.7) to assess the suppression ability of the seven isolates by determining the expression of inflammatory factors *in vitro*. Furthermore, *in vivo* tests were carried out by oral administration of *S. thermophilus* 19, which were selected by *in vitro* test, to mice in an LPS-induced inflammation model. The gut microbiota alterations and the levels of several inflammatory factors in various organs were determined to evaluate the influence of *S. thermophilus* 19. We hypothesize that supplementation of diets with probiotics could protect the visceral organs after sepsis by reducing inflammation through alterations of the gut microbiota.

## Materials and Methods

### Bacteria and Media

*L. plantarum* TW1-1, *Pediococcus acidilactici* XS40, *L. plantarum* DS45, *L. paracasei* LZU-D2, *L. delbruckii*, *L. casei* 18-10 and *Streptococcus thermophilus* 19 were all isolated from homemade yogurt, and *S. thermophilus* 19 was preserved at Guangdong Microbial Culture Collection Center (GDMCC) with No. 61312. Bacterial strains were cultured in De Man, Rogosa, and Sharpe (MRS; Beijing Solarbio Science & Technology, Beijing, China) growth medium with the exception of 19 and XS40, which were cultured in M17 growth medium (MRS; Beijing Solarbio Science & Technology, Beijing, China) supplemented with 1% lactose and MRS medium supplemented with 0.5% glucose. MRS and M17 agar medium (Beijing Solarbio Science & Technology, Beijing, China) were used to determine the CFU of the assayed probiotic strains.

### *In vitro* Evaluation of Inflammatory Factors Induced by Probiotics

The commercial immortal mouse macrophage cell line RAW264.7 was obtained from the American Type Culture Collection and was grown in Dulbecco’s Modified Eagle’s Medium (DMEM; Gibco, Gaithersburg, MD, United States), supplemented with 10% heat-inactivated fetal bovine serum (FBS) under a humidified 5% CO_2_ atmosphere at 37°C. In order to investigate the influence of strains, the cells were cultured in 12-well culture plates at 1 × 10^6^ cells/well. 200 μL bacterial strains were added into 5 mL MRS or M17 medium and incubated overnight (16 h). The cultures were obtained by centrifugation under 1,000 rpm for 3 min and washed with phosphate-buffered saline (PBS; pH 7.4), after which the cultures were diluted to an optical density (OD) of 0.3, re-suspended in PBS, and then 50 μL cultures were used to infect the RAW264.7 cells at the final multiplicity of infection (MOI), which was 1:100 (cells/bacteria). The plates were incubated for 6 h at 37°C under a 5% CO_2_ atmosphere and samples were collected to assess the levels of inflammatory factors by qRT-PCR. PBS without bacteria was used as negative control.

### CCK-8 Assay

The viability of RAW264.7 was detected by CCK-8 assay. Cells were seeded into 96-well plates with 1 × 10^3^ cells/well and treated with different doses of *S. thermophilus* 19 strains for 6 h, then 100 μL CCK-8 agent was added to medium and incubated for 1 h. OD450 nm was read with a microplate reader. Each experiment was performed 6 times independently.

### Animals and Sepsis Model

The 7-14-week-old male BALB/c (H-2D^d^) mice (average weight 20 *g*) used in this study were originally purchased from the Experimental Animal Center of The Fourth Military Medical University and were bred in our facility under specific-pathogen-free conditions. All animals were maintained under a 12 h light/dark cycle. In order to investigate the effect of LPS and *S. thermophilus* 19 on the survival rate, mice were administered different doses of LPS by intraperitoneal injection and treated with it for 2 days. Each group had 10 male mice. To investigate the influence of *S. thermophilus* 19 on sepsis, mice were administered 1 mg/kg LPS by intraperitoneal injection, with a second dose administered 4 days after the first injection. Mice received 0.3 ml *S. thermophilus* 19 (1 × 10^9^ CFU/ml) or PBS once every other day. Mice were anesthetized with isoflurane after treatment for 3 days (the total treatment time was 1 week). Blood in mice was obtained by cardiac blood collection under anesthesia. Then the lung, small intestine, liver, and kidney tissues were collected and divided into two parts. One was used for histology which was fixed in 4% paraformaldehyde (PFA). The other part, weighing around 200 mg, was stored at -80°C for mRNA analysis and then added to 2 ml Trizol. Feces were obtained from cecum and stored at liquid nitrogen for gut microbiota analysis. The details of the experimental design are shown in Table 1. All procedures and protocols used in this study conform to the institutional guidelines and were approved by the Ethics Committee of the Fourth Military Medical University.

**TABLE 1:** Experimental design.

**Group**	**Treatment groups (*n* = 8)**	**Gavage**
1	Control	PBS
2	LPS only	PBS
3	LPS + *S. thermophilus* 19	PBS + *S. thermophilus* 19
4	*S. thermophilus* 19 only	PBS + *S. thermophilus* 19

*LPS: 1 mg/ml; S. thermophilus 19: 1 × 10^9^ CFU/ml once every other day in 0.3 ml PBS. Mice received LPS (1 mg/kg) through intraperitoneal injection. Mice received PBS and 19 via gavage.*

### Weight, Water, and Food Intake Measurements and Sampling

Body weight, water and food intake, and stool appearance were documented for all groups of mice every other day throughout the experiment. After 1 week, livers, kidneys, lungs, and small intestines were collected from each mouse and were divided into triplicate samples, with one stored in liquid nitrogen, a second stored in RNAiso Plus for RNA extraction, and the third fixed in 4% (w/v) PFA at 4°C for later histological analysis.

### Histology of Different Tissues

After the animals were sacrificed, different tissue samples were collected. After fixation in 4% PFA, tissue samples were embedded in paraffin and serially cut into 7-mm thick sections. Tissue slides were stained with hematoxylin and eosin (H&E) for histological analysis. The histopathologic scores of different tissues were evaluated through different scores systems (Gloor et al., 2000; Ji et al., 2018; Ko et al., 2020; Yang et al., 2020).

### Microbial DNA Extraction and Illumina MiSeq Sequencing

Microbial DNA was extracted from the samples using an E.Z.N.A.^®^ Stool DNA Kit (Omega BioTek, Norcross, GA, United States) according to manufacturer’s protocols, and the DNA samples were assessed via PCR with the universal 16S rRNA primers 27F/1492R in our own lab. The DNA concentration and integrity were determined by electrophoresis on 1% agarose gels containing ethidium bromide and spectrophotometrically using an EPOCH instrument (BioTek). After confirmation, the DNA was lyophilized and sent for Illumina MiSeq sequencing and data analysis.

The gut microbiota compositions of mice were assessed via Illumina MiSeq sequencing (Genergy Biotech) targeting the V3-V4 region of the bacterial 16S ribosomal RNA gene using the primers 341F (5′-CCTACGGGNGGCWGCAG-3′) and 785R (5′-GACTACHVGGGTATCTAATCC-3′), with an eight-base barcode sequence unique to each sample. The amplicons were extracted from 2% agarose gels and purified using an AxyPrep DNA Gel Extraction Kit (Axygen Biosciences, Union City, CA, United States) according to the manufacturer’s instructions and were subsequently quantified using a QuantiFluor^TM^-ST instrument (Promega, United States). The purified amplicons were pooled in equimolar ratios and paired-end sequenced (2 × 300) on an Illumina MiSeq platform according to standard protocols. The raw reads were deposited at the NCBI Sequence Read Archive (SRA) database. Operational taxonomic units (OTUs) were clustered with a 97% similarity cutoff using UPARSE (version 7.1 http://drive5.com/uparse/), and chimeric sequences were identified and removed using UCHIME. The taxonomy of each 16S rRNA gene sequence was analyzed using RDP classifier^1^ against the SILVA (SSU123) 16S rRNA database using a confidence threshold of 70%. The taxonomy of each ITS gene sequence was analyzed using Unite classifier^2^. The function genes of gut microbiota were predicted through the Tax4Fun package of R, which has proven to be highly consistent with the results generated from metagenomic sequencing approaches. Based on the association relationship between the taxonomy information and prokaryotic database calculated previously, the OTU classification matrix annotated by SILVA was turned into the KEEG matrix. Then, the abundance of genes involved in KEEG pathways was standardized according to the copies of corresponded bacterial 16S rRNA genes. The function of gut microbiota was subsequently predicted by KEGG database. The correlation between the inflammatory factors and bacteria were investigated by R (version: 3.6.1) using spearman correlation analysis. The calculated data were submitted to corrplot R package to generate the correlation plot with the Significant confidence interval of 0.95.

### Quantitative RT-PCR for Inflammatory Factor Determination

Total RNA was extracted from different tissues using RNAiso Plus (Takara, Dalian, China) and was subsequently reverse transcribed into cDNA using PrimeScript^TM^ RT Kit (Takara, Dalian, China) according to the manufacturer’s protocol. The expression of inflammatory factor-related genes was analyzed using SYBR^®^ PremixEx Taq^TM^ II and the Bio-Rad CFX system. For real-time PCR, the reaction mixtures contained 1 μL cDNA, 0.4 μL of each primer (10 mmol^–1^), 5 μL of SYBR green PCR Master Mix, and distilled water to reach a final reaction volume of 10 μL. The Taq DNA polymerase was activated at 95°C for 10 min, followed by 40 cycles of 95°C for 15 s, 60°C for 30 s, and 72°C for 30 s. Quantitative RT-PCR data were normalized to the expression of the housekeeping gene β-actin using the 2^–Δ^ ^Ct^ method. Primers used in this study are shown in Table 2.

**TABLE 2:** Primers used in this study.

**Primer**	**Sequence (5′-3′)**
β-actin	GTACGCCAACACAGTGCTG/CGTCATACTCCTGCTTGCTG
IL-1β	GCTTCAGGCAGGCAGTATC/AGGATGGGCTCTTCTTCAAAG
TNF-α	AGAGCTACAAGAGGATCACCAGCAG/TCAGATTTACGGGTCAACTT CACAT
IL-6	GAGGATACCACTCCCAACAGACC/AAGTGCATCATCGTTGTTCATACA

### Quantification and Statistical Analysis

Graphpad Prism was used for graphical presentation. For the *in vitro* and mice experiments, we used rank sum test to calculate the *p*-value. For gut microbiota, non-parametric statistical analysis was used. Wilcoxon test was applied for two independent samples, while Kruskal–Wallis test was applied for intergroup analysis. *P* < 0.05 was considered statistically significant. The number of biological replicates (n) and the number of independent experiments is indicated in the figure legends.

## Results

### *S. thermophilus* 19 Decrease the Expression of Inflammatory Factors *in vitro*

**FIGURE 1:**
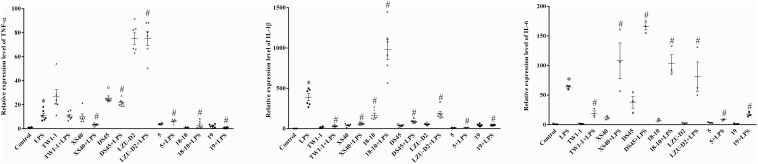
The expression of inflammatory factors (IL-1β, TNF-α, and IL-6) in LPS-treated, strains-LPS treated, and untreated RAW264.7 cells (*n* = 3). Error bars represents SEM. ^∗^*P* < 0.05, Compared with control group. ^#^*P* < 0.05, Compared with LPS group.

A co-culture system of strains and mouse macrophage RAW264.7 cell was developed to assess the influence of the candidate strains on the inflammatory response. Seven isolates, including *L. plantarum* TW1-1, *P. acidilactici* XS40, *L. casei* 18–10, *L. plantarum* DS45, *L. paracasei* LZU-D2, *L. delbruckii* 5, and *S. thermophilus* 19, were selected for this *in vitro* test. The results showed that LPS treatment significantly increased the expression of inflammatory factors (IL-1β, TNF-α, and IL-6) compared to the control group. All the co-culture systems of strain and LPS-treated cells presented lower expression levels of IL-1β than LPS group, except *L. casei* 18–10. Additionally, the co-culture of *L. delbruckii* 5, *L. casei* 18–10, and *S. thermophilus* 19 also reduced the expression of TNF-α, *L. plantarum* TW1-1, *L. delbruckii* 5, and *S. thermophilus* 19 and decreased the expression of IL-6 in LPS-treated cells (Figure 1). As a result, *S. thermophilus* 19 was chosen for further study due to its optimal properties. We also made sure that *S. thermophilus* 19 had no effect on cell viability before carrying out the *in vivo* test. We found that *S. thermophilus* 19 had no effect on cell viability under doses up to 1 × 10^10^ CFU/ml (Supplementary Figure 1).

### *S. thermophilus* 19 Effectively Alleviated Inflammation Induced by Sepsis

**FIGURE 2:**
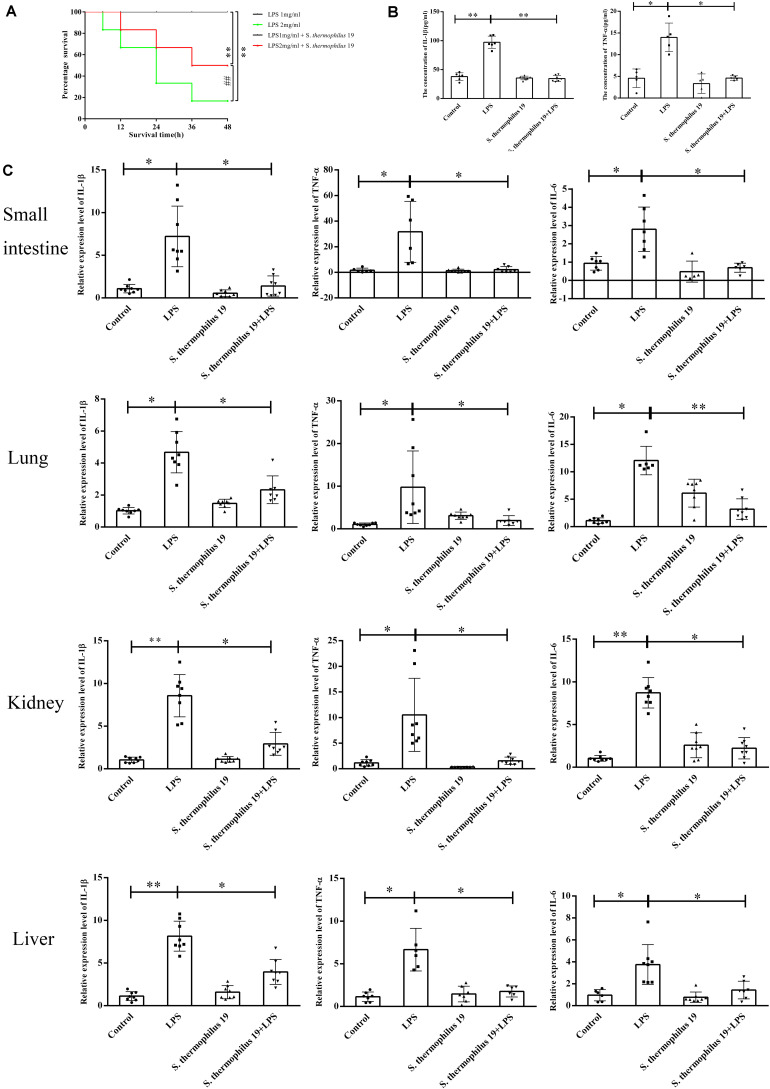
*S. thermophilus* 19 alleviate the inflammatory reaction caused by LPS-induced sepsis. **(A)** Survival rates of mice with or without *S. thermophilus* 19 treatment after 48 h stimulation with different dose of LPS (*n* = 10). **(B)** Levels of IL-1β and TNF-α in blood were determined using commercial ELISA kits (*n* = 8). **(C)** *S. thermophilus* 19 intervention resulted in decreased inflammation in the small intestine, lung, liver, and kidney (*n* = 8). Error bars represents SEM. ^∗^*P* < 0.05 and ^∗∗^*P* < 0.01.

Firstly, the influence of different doses of LPS on mice survival rate was investigated. All mice died when the concentration of LPS exceeded 2.5 mg/kg, even in mice administered *S. thermophilus* 19 (data not shown). However, 55% of mice survived when administered strain 19 together with 2 mg/ml LPS, whereas only 20% of mice survived when administered the 2 mg/kg LPS without bacteria (Figure 2A). All mice treated with 1 mg/kg LPS with or without strain 19 survived (Figure 2A). Thus, 1 mg/kg LPS was chosen to investigate the influence of *S. thermophilus* 19 on gut microbiota and inflammation in sepsis mice. Mice treated with LPS (1 mg/kg) lost approximately 10% of their body weight at 48 h after injection, while the weight of untreated mice did not drop (Supplementary Figure 2A). Total food and water intake and the animal health conditions were recorded. LPS-treated mice with or without *S. thermophilus* 19 both exhibited a reduction in total drinking water and rat chow intake, as did mice administrated *S. thermophilus* 19 alone (Supplementary Figures 2B,C). However, mice administrated *S. thermophilus* 19 alone showed no changes in body weight (Supplementary Figures 2B,C). Hence, the reduction in body weight of the LPS-treated mice could be explained by the LPS-induced inflammation causing a reduction in food and drinking water intake, while *S. thermophilus* 19 could alleviate inflammation to promote the recovery in body weight.

The expression of TNF-α and IL-1β increased 2-fold and 2.5-fold, respectively, after LPS treatment, while it was reduced to that observed in the control group in mice administered *S. thermophilus* 19 (Figure 2B). In contrast, no significant difference was observed in the serum levels of IL-1β and TNF-α in mice administered *S. thermophilus* 19 without LPS treatment, demonstrating that *S. thermophilus* 19 has no effect on the normal mice.

The inflammation response of kidneys, small intestines, livers, and lungs of each mouse after LPS and strain 19 treatment was investigated. LPS treatment dramatically increased the expression of IL-1β, IL-6, and TNF-α in all tissues while they were effectively rescued in the mice administrated *S. thermophilus* 19 (Figure 2C).

**FIGURE 3:**
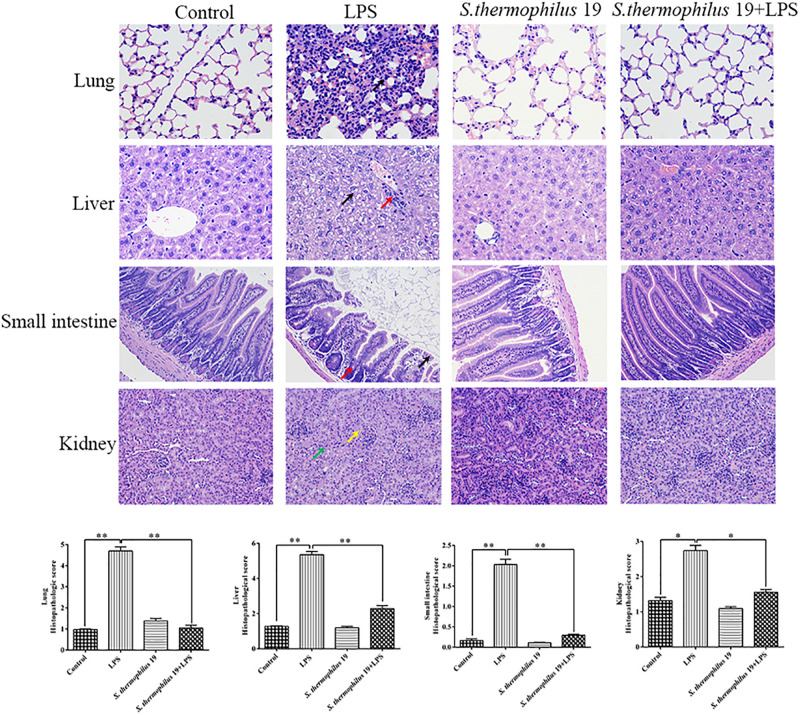
Hematoxylin and eosin staining and histopathologic scores of lung, liver, small intestine, and kidney tissues from different groups. Black arrow in lung sections represent inflammatory cells infiltrating the alveolar walls. In liver sections, black arrow represent that the cytoplasm is loose or vacuolated and red arrow indicates numbers of lymphocytes surrounding the portal area. Black arrow represents epithelial cells exfoliate and red represents inflammatory cells infiltrating in small sections. In kidney sections, yellow arrow represents tubules that are structurally unclear and green arrow indicates a number of tubule epithelial cells swollen and loose. Sections were examined and photographed under a microscope (*n* = 8). ^∗∗^*P* < 0.01 and ^∗^*P* < 0.05.

Hematoxylin and eosin staining revealed that, compared with liver sections of control group mice, significant congestion of veins and hepatocyte necrosis was observed in the LPS-treated mice, and the loss of intact liver plates and hepatocyte vacuolization was observed (Figure 3). In pulmonary sections, drastic destruction of alveolar structures was detected in the LPS-treated mice, and the effusion in alveoli in these mice was markedly more severe than that observed in the control group mice. Furthermore, tissue infiltration by inflammatory cells was substantially higher in LPS-treated mice than in the control group mice (Figure 3). In small intestine sections, the intestinal villi in the LPS group were short. A small amount of mucosal epithelial cells shed and an amount of inflammatory cells infiltration in the epithelium was also detected. Moreover, the intestinal glands were loosely arranged. The local tubules of kidney sections in LPS-treated mice were not clear (black arrow). Moreover, numbers of epithelial cells in the tubules were swollen and the cytoplasm was loose. The renal tubules were narrow. All tissues histopathologic scores in the LPS group significantly increased (Figure 3). Co-treatment with *S. thermophilus* 19 resulted in the restoration of a close-to-normal appearance of all tissues. Moreover, *S. thermophilus* 19 treatment did not affect all tissue sections of normal mice (Figure 3).

### *S. thermophilus* 19 Administration Significantly Changed the Community Structure of the Gut Microbiota

**FIGURE 4:**
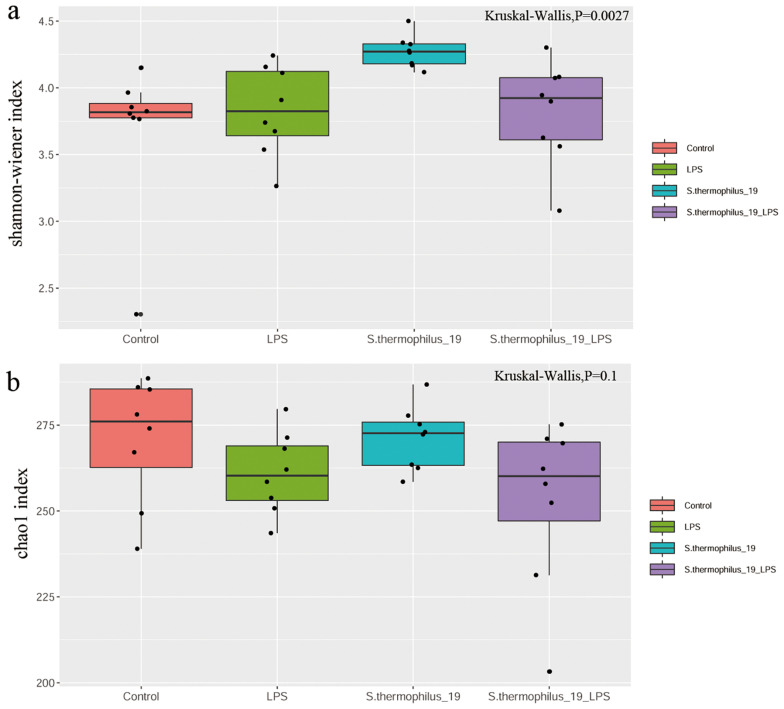
LPS induces significant impact on microbiota composition. **(A)** Shannon index. **(B)** Chao1 index. (*n* = 8/group).

The cecal feces were collected for gut microbiota determination. The results showed the microbial diversity (Shannon-wiener index) were significantly different among groups. *S. thermophilus* 19 administration significantly increased the α-diversity of the gut microbiota either in normal or LPS-treated mice. The microbial richness (Chao1 index) also decreased after LPS treatment, but the supplementation of *S. thermophilus* 19 seems have no significant effect on the richness of bacterial community. (Figures 4A,B).

### Administration of *S. thermophilus* 19 Altered the Gut Microbiota Composition

**FIGURE 5:**
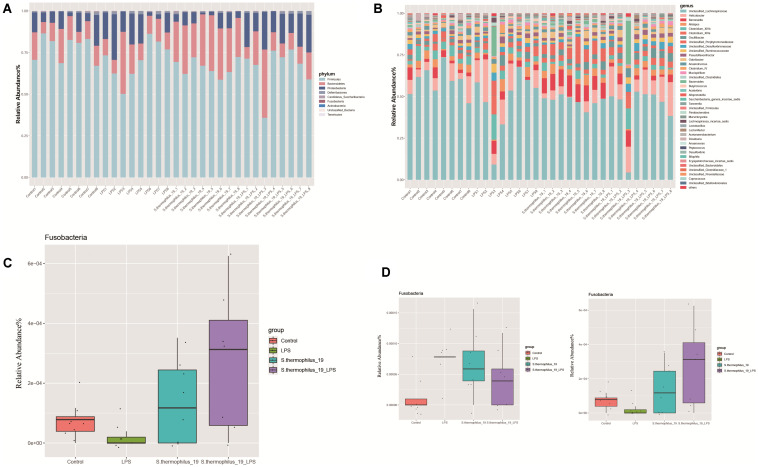
*S. thermophilus* 19 has a significant impact on microbiota composition (*n* = 8). **(A)** The change of gut microbiota at phylum level. **(B)** The change of gut microbiota at genus level. **(C)** The details of the change of *Fusobacteria* in different groups. **(D)** The details of the change of *Flavonifractor* and *Fusobacterium* in different groups.

Gut microbiota composition of the mice from four groups were analyzed at phylum and genus levels (Figures 5A,B). Among all identified phyla, the relative abundances of Fusobacteria in four groups were markedly different (*P* = 0.037). LPS treatment decreased the *Fusobacteria* and the administration of strain 19 increased it dramatically (Figure 5C). At a genus level, most of the microbiota were dominated by unclassified *Lachnospiraceae*, *Helicobacter*, and *Barnesiella*, with an exception of two LPS-treated mice, which were dominated by *Bacteroides* and *Clostridium* (Figure 5B). This may indicate mice also have different enterotypes, just like human beings. *Flavonifractor* and *Fusobacterium* were the genera significantly different among groups (*P* = 0.009 and 0.038, respectively). *Fusobacterium* significantly decreased after LPS treatment and its abundance raised after strain administration, while *Flavonifractor* showed an opposite trend (Figure 5D).

**FIGURE 6:**
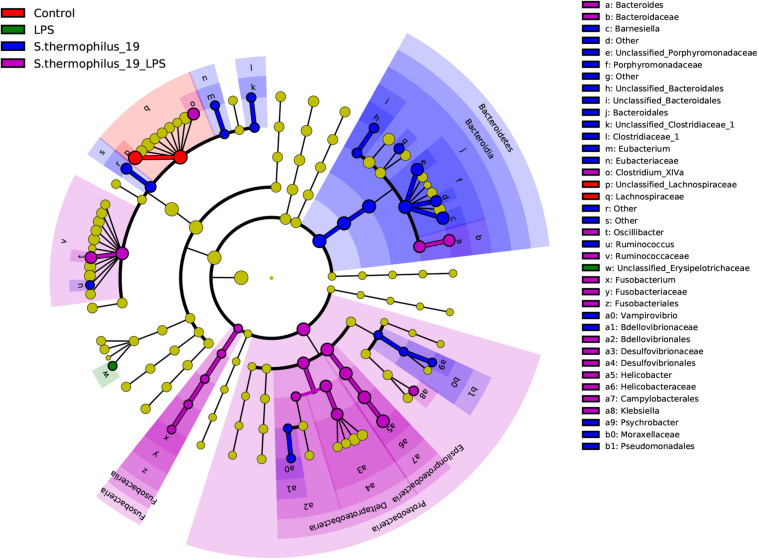
Linear discriminate analysis effect size analyzed the differentially abundant features in the four groups. The cladograms represented here indicate the bacterial taxa that are significantly different between the four groups being compared. This analysis helps to identify a first selection of differential bacterial taxa in the considered groups.

Differentially abundant features in the four groups were determined using Linear discriminate analysis effect size (LEfSe), which is an algorithm for characterizing genomic features most likely to explain differences between groups. Species in the *Bacteroidia*, *Proteobacteria*, and *Fusobacteria* may be regarded as the main responders of the oral administration of strain 19 on normal and LPS-treated mice (Figure 6). The correlation between the bacterial composition with the inflammatory factors was also analyzed by R (version: 3.6.1) using spearman correlation analysis. We found that *Fusobacterium* presented correlations with TNF factors, as well as *Flavonifractor*. These two bacterial genera were the key bacteria discovered in our study and this result indicted that *Fusobacterium* and *Flavonifractor* may have functioned in alleviating the inflammation reaction by TNF regulation. In addition, *Clostridium* group, which was reported to be an important butyrate-producing bacterial group in the gut microbiota, were correlated with IL-6. It can be inferred that the structure of Clostridium group may also contribute to the effectiveness of *S. thermophilus* 19 (Supplementary Figure 4).

### The Function of Gut Microbiota Was Specifically Altered After the Administration Oral Probiotics

Subsequently, the function genes of type strain of the different species were used to predict how the altered community structure of the gut microbiota affects its function. The abundance of the genes involved in primary bile acid biosynthesis and secondary bile acid biosynthesis pathways were decreased in mice treated with LPS and *S. thermophilus* 19 compared to the LPS-treated mice, which may contribute to the attenuate inflammation reactions (Supplementary Figure 3). The genes involved in DNA replication and repair, metabolism of cofactors and vitamins, xenobiotics biodegradation and metabolism, energy metabolism, and lipid metabolism were significantly different among the four groups. However, the metabolism analysis in this study is only based on the genomes of type strain from identified species, so it could not reflect the true microbial function of the mice gut in this experiment. Hence, further studies and more advanced sequencing methods, like metagenomic sequencing, should be used to explore the alteration of the microbial metabolic networks induced by LPS and strain administration.

## Discussion

In this study, the MiSeq 16S rDNA sequencing approach was utilized to assess how *S. thermophilus* 19 modulates the host fecal microbiota and inflammatory response in an LPS-induced mouse sepsis model. The results indicated that the administration of strain 19 could effectively rescue the over expression of IL-1β, IL-6, and TNF-α induced by LPS treatment, which may result from the alteration of gut microbiota pattern, especially the abundance of species from Fusobacteria phylum.

The mice model used in this study, the Endotoxin/Lipopolysaccharide Model, is one of the most common sepsis models, (Murando et al., 2019). LPS is well-known as an important mediator of sepsis, with excessive LPS resulting from uncontrolled infection even causing septic shock (Wang et al., 2015b). Mice in this study manifested biochemical and physiological changes after receiving 1 mg/kg LPS. We observed that LPS significantly upregulates the expression of genes involved in inflammation, especially in the livers, lungs, kidneys, and small intestines of mice, which indicated that multiple organ dysfunction was caused after LPS injection and this coordinated with the outcomes of the sepsis model in the previous study. In addition, for mice treated with 2 mg/ml LPS, the survival rate increased dramatically in the mice administrated with *S. thermophilus* 19, which indicated this strain is able to protect mice from the damage caused by LPS.

In recent years, it was discovered that inflammation and infection were accompanied by an imbalance in the gut microbiota (Khosravi et al., 2014). Inflammatory response mounted against microfloral bacteria, leading to a perpetuation of the inflammation and gut barrier dysfunction (Schuijt et al., 2016). Previous studies showed that the inflammation reaction of the host was associated with the shifts of intestinal *Firmicutes* to *Bacteroidetes* ratio, as well as the reduction of microbiota diversity (Zaborin et al., 2014; Ojima et al., 2016). However, the model of these studies had many uncertain factors when regarding the variability and temporal nature of sepsis-induced dysbiosis. Thus, we used an LPS-induced sepsis model to investigate the changes in gut microbiota composition to eliminate the influence of other factors. In this study, the microbiota diversity presented significant differences among groups, and the α-diversity was increased by the administration of strain 19 which may link it with the alleviation in inflammation. For the microbiota composition, genera *Fusobacterium* and *Flavonifractor* were found to be significantly decreased during the development of sepsis and raised again after the administration of *S. thermophilus* 19, which demonstrated these two genera were the key bacteria that may function in the mice gut microbiota. The abundance of *Flavonifractor* in colorectal cancer, inflammatory bowel diseases, high-fat diet rats, and acute liver injury rat models was significantly increased (Ai et al., 2019; Armstrong et al., 2019; Meng et al., 2019). In addition, although the composition of gut bacteria in mice treated with *S. thermophilus* 19 and that of the control group differed, the expression of inflammation-associated factors in these mice did not significantly differ. What’s more, the results also inferred that mice treated with LPS and *S. thermophilus* 19 exhibited changes in the function of bile acid biosynthesis, which could be important pathways involved in inflammation regulation, as previous studies have indicated (Wang et al., 2015a; Sonnenburg and Backhed, 2016). Probiotics are live microbial food supplements or bacterial components that have been shown to have beneficial effects on human health (Sanchez et al., 2017). Additionally, probiotics are often used to treat inflammation-related diseases, such as inflammatory bowel disease, allergic diseases, and acute gastroenteritis (Gareau et al., 2010; Fiocchi et al., 2015; Schnadower et al., 2018). *S. thermophilus* is a probiotic that has been used in the treatment of some diseases in recent research. Probiotics containing *S. thermophilus* KB19 significantly increased betaine plasma levels in chronic kidney disease.

*Streptococcus thermophilus* has also been used to help prevent the development of insulin resistance in previous research (Asemi et al., 2013). *S. thermophilus* also has antioxidant properties through scavenging reactive oxygen radicals and shows immunomodulatory effects by stimulating the gut immune system (Del Carmen et al., 2014). In our study, we first explored the effect of *S. thermophilus* in LPS-induced sepsis mouse models. The administration of *S. thermophilus* 19 significantly decreased the level of inflammatory factors in sepsis mice and did not trigger any inflammation deviation or any other obvious harmful side effects in normal mice, suggesting that it could be used safely for humans and had the potential of application for sepsis intervention in the future.

In summary, we demonstrated that *S. thermophilus* 19 can alleviate inflammation both *in vivo* and *in vitro*. This probiotic reduced the levels of inflammatory factors in the LPS-induced sepsis mice model, which may occur through multiple targets. For instance, *S. thermophilus* 19 can remodel the dysbiosis of gut microbiota after intraperitoneal injection of LPS by enriching the beneficial bacteria and resisting the pathogenic bacteria. Also, it has the potential for altering the function of intestinal microbes by reshaping the composition of the microbiota. These studies showed that *S. thermophilus* 19 may be applied in the treatment of not only sepsis but also other systemic inflammatory diseases, such as inflammatory bowel disease, systemic inflammatory arthritis, multiple sclerosis, and so on. Furthermore, we provided a novel potential treatment target for sepsis and other systemic inflammatory diseases.

## Data Availability Statement

The datasets presented in this study can be found in online repositories. The names of the repository/repositories and accession number(s) can be found below: https://www.ncbi.nlm.nih.gov/, PRJNA661124.

## Ethics Statement

The animal study was reviewed and approved by Ethics Committee of the Fourth Military Medical University.

## Author Contributions

XZ and DH designed and supervised the study. FH, GW, and YZ performed the experiments and wrote the manuscript. HZ analyzed the data. SH and XL conducted the animal trial and sample collection. WC and JL conducted physiological data analysis and edited the manuscript. WZ helped with animal experiments and provided critical experimental materials. All authors contributed to the article and approved the submitted version.

## Conflict of Interest

The authors declare that the research was conducted in the absence of any commercial or financial relationships that could be construed as a potential conflict of interest.

## Footnotes

1 http://rdp.cme.msu.edu/

2 https://unite.ut.ee/index.php

## Supplementary Material

Supplementary Figure 1 The effect of different doses of *S. thermophilus* 19 on cell viability was detected by CCK8 assay after co-culture of 6 h (*n* = 6). Error bars represent SEM. ^∗^*P* < 0.05, ^∗∗^*P* < 0.01, Compared with PBS group.

Supplementary Figure 2 The influence of *S. thermophilus* 19 and LPS on body weight, total rat chow, and drinking water intake. **(A)** Body weight change and relative weight change (*n* = 8/group). **(B)** Total rat chow intake. **(C)** Total drinking water. ^∗^*P* < 0.05, Compared with PBS group.

Supplementary Figure 3 The presence of *S*. *thermophilus* 19 induces changes in gut microbiota function after LPS treatment (*n* = 8/group). Statistical comparison was performed by first testing normality using Kruskal–Wallis/Wilcoxon rank-sum test. Error bars represent SEM.

Supplementary Figure 4 The correlation between the bacterial composition with the inflammatory factors (*n* = 8/group). The calculated data were submitted to corrplot R package to generate the correlation plot with the Significant confidence interval of 0.95.
