# Supplementary figures and images for: Streptococcus thermophilus Attenuates Inflammation in Septic Mice Mediated by Gut Microbiota
Source: Front Microbiol. 2020 Dec 15;11:598010. doi: 10.3389/fmicb.2020.598010 (PMC7769777; doi:10.3389/fmicb.2020.598010)

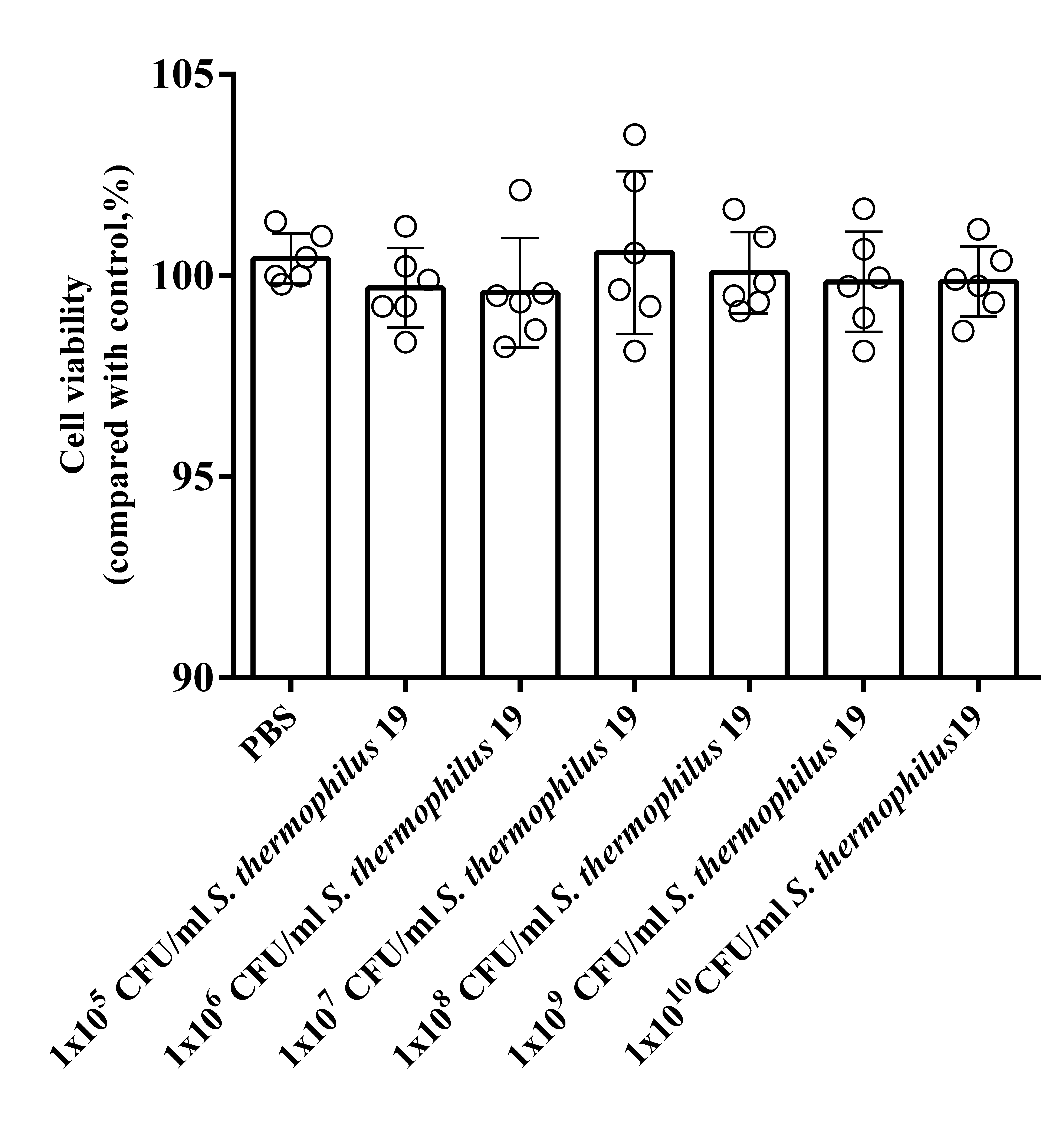

Supplement: Supplementary Figure 1 — The effect of different doses of S. thermophilus 19 on cell viability was detected by CCK8 assay after co-culture of 6 h (n = 6). Error bars represent SEM. ∗P < 0.05, ∗∗P < 0.01, Compared with PBS group. [file Image_1.TIF]

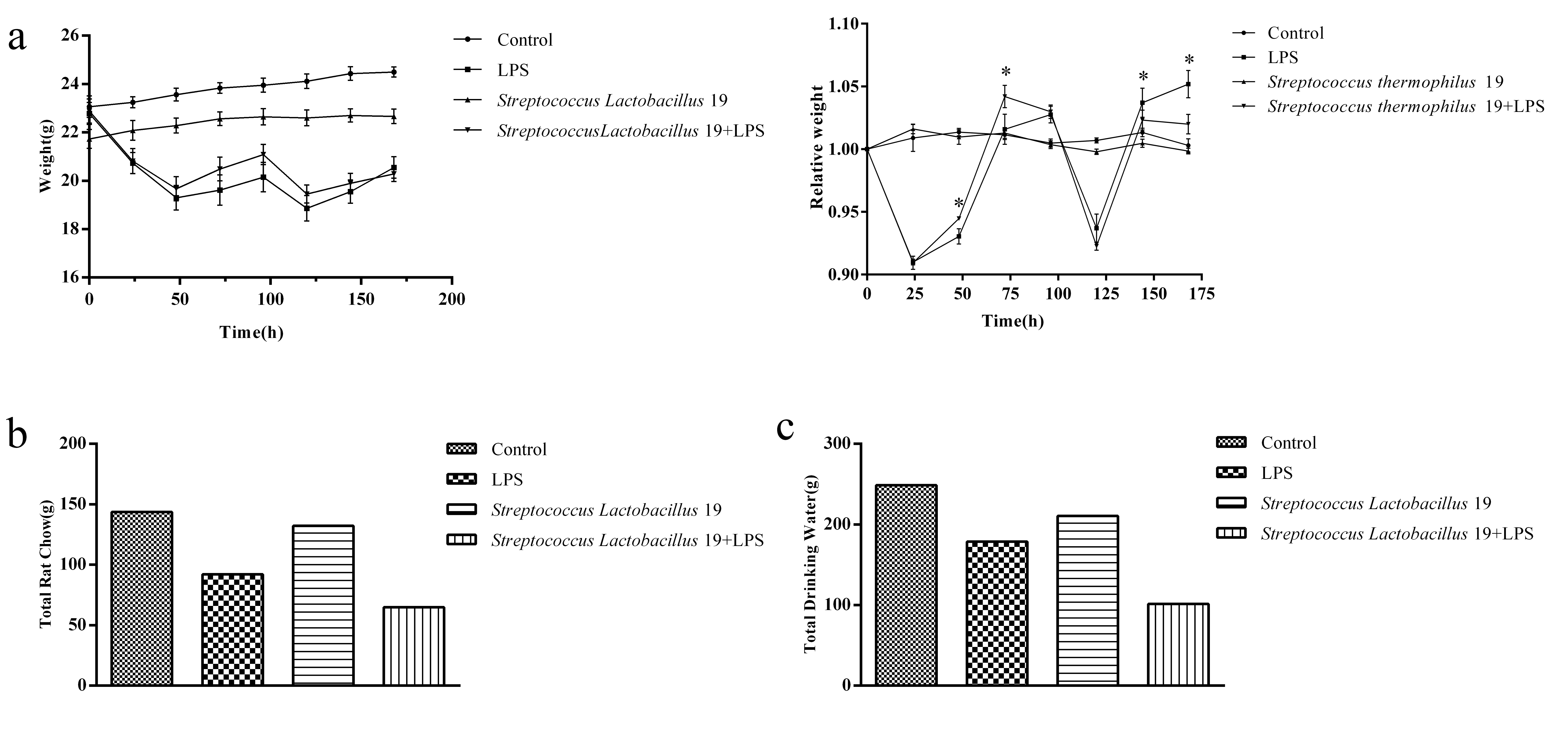

Supplement: Supplementary Figure 2 — The influence of S. thermophilus 19 and LPS on body weight, total rat chow, and drinking water intake. (A) Body weight change and relative weight change (n = 8/group). (B) Total rat chow intake. (C) Total drinking water. ∗P < 0.05, Compared with PBS group. [file Image_2.TIF]

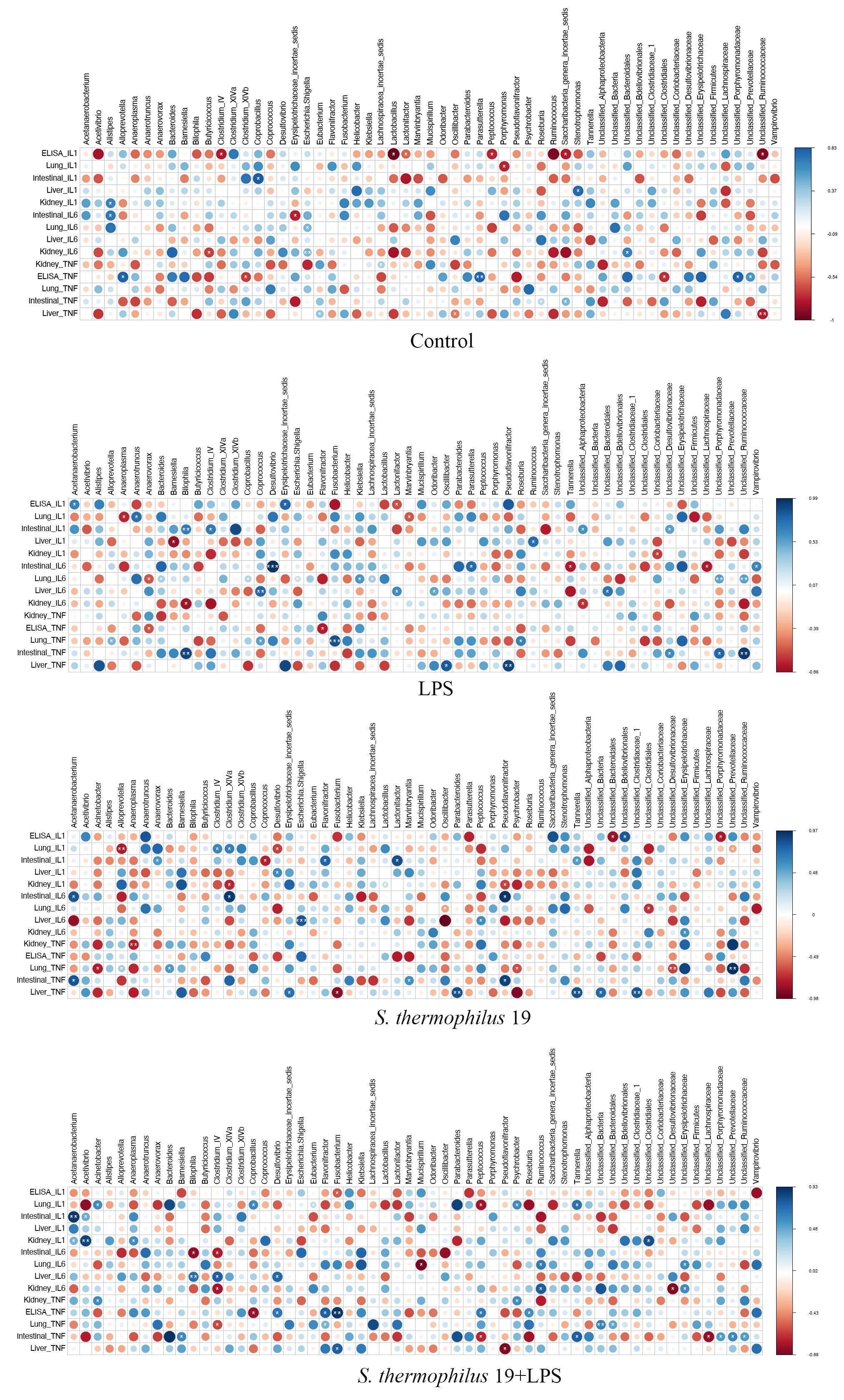

Supplement: Supplementary Figure 4 — The correlation between the bacterial composition with the inflammatory factors (n = 8/group). The calculated data were submitted to corrplot R package to generate the correlation plot with the Significant confidence interval of 0.95. [file Image_4.TIF]
